# ShenQiGan Extract Repairs Intestinal Barrier in Weaning-Stressed Piglets by Modulating Inflammatory Factors, Immunoglobulins, and Short-Chain Fatty Acids

**DOI:** 10.3390/ani15152218

**Published:** 2025-07-28

**Authors:** Rongxia Guo, Chenghui Jiang, Yanlong Niu, Chun Niu, Baoxia Chen, Ziwen Yuan, Yongli Hua, Yanming Wei

**Affiliations:** College of Veterinary Medicine, Gansu Agricultural University, Lanzhou 730070, China; 15214043697@163.com (R.G.); jch18215171914@163.com (C.J.); 18419472665@163.com (Y.N.); niuchun1999@163.com (C.N.); cbx961031@163.com (B.C.); yuanziwen_77@163.com (Z.Y.)

**Keywords:** weaning stress, piglet, ShenQiGan extract, intestinal barrier

## Abstract

Weaning stress damages the intestines and disrupts the intestinal barrier in piglets, which significantly impacts the pig farming industry’s economy. We aimed to examine the effects of ShenQiGan extract (CAG) on intestinal barrier function and explore the underlying molecular mechanisms in stress-challenged weaned piglets. The experimental design involved 80 weaned piglets aged 28 days (with an average body weight of 7.78 ± 0.074 kg) that were randomly allocated into four groups: Control, LCAG (0.1% CAG), MCAG (0.5% CAG), and HCAG (1.0% CAG). After a 28-day trial period, the growth performance and incidence of diarrhea in piglets were evaluated. CAG increased the average daily gain of weaned piglets, reduced the feed-to-gain ratio, and decreased the incidence of diarrhea. It significantly lowered serum inflammatory cytokine levels while elevating immunoglobulin levels. The supplement notably enhanced concentrations of acetic acid, propionic acid, butyric acid, and isobutyric acid. Furthermore, CAG demonstrated intestinal morphology restoration and upregulation of tight junction proteins and MUC2 protein expression in jejunum. At the mRNA level, it significantly upregulated the expression of Occludin, Claudin1, and MUC2 genes. CAG improves growth performance and mitigates diarrhea in weaned piglets by enhancing intestinal barrier integrity, modulating systemic inflammatory responses, elevating immunoglobulin levels, and promoting short-chain fatty acids (SCFAs) production in the cecum.

## Simple Summary

This study explores CAG, a herbal formula, to mitigate weaning stress in piglets, a major industry issue causing intestinal dysfunction and growth delays. Unlike conventional antibiotics/single-target approaches, CAG is evaluated as a multi-functional intervention. The work innovatively integrates growth metrics, molecular assays, and cecal short-chain fatty acids profiling to holistically assess CAG’s effects on intestinal barrier repair, systemic inflammation, immunoglobulin regulation, and microbial metabolism. This systems-level analysis advances understanding of phytogenic additives in livestock nutrition, offering a novel strategy to address weaning-associated challenges.

## 1. Introduction

The weaning of piglets represents a critical phase in their developmental trajectory. The process involves the separation of piglets from their mothers, exposure to new environmental conditions, alterations in feeding regimens, and the administration of vaccinations, all of which can induce significant stress. This stress is associated with detrimental effects such as intestinal barrier damage, diarrhea, and growth retardation [1]. Empirical evidence shows that piglets usually lose about 100–250 g in body weight on the first day after weaning [2]. Furthermore, achieving a daily weight gain exceeding 227 g during the first week post-weaning correlates with a reduction of 6–10 days to reach market weight, compared to piglets that gain only 150 g per day. This early weight gain is a critical determinant of the total duration required to reach market weight, approximately 110 kg [3]. In this study, 28-day-old weaned piglets experienced a 10 h long-distance transport and had an 18% diarrhea rate in the first two weeks post-weaning [4], suggesting that weaning stress was caused. Nutritional intervention has emerged as an effective strategy to alleviate stress, promote intestinal health, and control pathogen infections. In recent years, there has been considerable attention on the incorporation of plant extracts, minerals, prebiotics, probiotics, vitamins, and amino acids into animal feeds [5,6,7,8]. Chinese herbal medicine has been recognized as a potential approach to address weaning stress in piglets. However, its application and widespread adoption have been hindered by insufficient mechanistic understanding and inconsistent therapeutic efficacy. This study therefore employs CAG (Chinese herbal compound additive) to elucidate its effects and mechanisms in alleviating weaning stress in piglets.

CAG, formulated through *Codonopsis pilosula*, *Astragalus membranaceus*, and *Glycyrrhiza uralensis*, was prepared following traditional Chinese veterinary medicinal principles of herbal compatibility with optimized botanical proportions. *Codonopsis pilosula* (Dangshen) demonstrates vital energy (Qi) tonification with specific tropism for lung and spleen meridians [9,10,11,12,13]. *Astragalus membranaceus* (Huangqi) exhibits Yang-ascent pharmacodynamics, resolving damp-heat through diuretic regulation. *Glycyrrhiza uralensis* (Gancao) serves as a harmonizing mediator in herbal compatibility [11,14,15,16,17]. These botanicals constitute the foundational elements of the classical Decoction for Strengthening the Middle Warmer and Benefiting Vital Energy (Buzhong Yiqi Decoction), designed for middle warmer fortification and Qi replenishment. Three Chinese herbal medicines have a synergistic effect, while a single Chinese herbal medicine exhibits an additive effect. Their synergistic interaction manifests through three-dimensional therapeutic effects: potentiation of Qi circulation via Yang elevation, splenic function activation, and pathological correction of Qi deficiency syndrome. Phytochemical profiling reveals CAG’s principal bioactive constituents—heteropolysaccharides, flavonoid glycosides, essential amino acids, and micronutrients—which collectively mediate antioxidant defense mechanisms and immunomodulatory responses in biological systems [18,19,20,21,22,23].

The intestinal barrier represents a complex biological system encompassing an epithelial cell layer, protective mucus coating, resident immune components, symbiotic microbial communities, and their metabolic byproducts [24]. Notably, SCFAs fulfill dual critical functions in this ecosystem, serving as the primary metabolic fuel for approximately 80% of intestinal epithelial cells while simultaneously supporting barrier defense mechanisms. Current research indicates these microbial-derived metabolites contribute to intestinal homeostasis through immunomodulation of mucosal-associated immune cells and reinforcement of epithelial structural continuity [25]. From a molecular perspective, maintenance of barrier integrity depends on adequate expression levels of specialized junctional proteins (including Claudin-1, ZO-1, and Occludin) along with sufficient production of the mucus-forming glycoprotein MUC2, as demonstrated through multiple experimental models [26]. Therefore, enhancing barrier integrity to improve gut health is an effective strategy to alleviate stress in weaned piglets.

This study sought to establish the optimal dosage of CAG by evaluating its influence on growth performance, diarrhea incidence, serum inflammatory markers, and antioxidant status in weaned piglets under stress. Upon identifying the most effective dose, subsequent analyses focused on intestinal barrier integrity and cecal SCFAs concentrations. These investigations aimed to clarify CAG’s protective mechanisms on the gut barrier. Ultimately, this research seeks to address intestinal barrier dysfunction caused by weaning stress in piglets and to enhance economic efficiency.

We therefore hypothesize that CAG stimulates butyrogenic microbiota, increasing butyrate synthesis. This attenuates systemic inflammation viaTLR4/NF-κB suppression (reducing IL-1β,TNF-α), while elevating immunoglobulins (IgA, IgM) to restore intestinal barrier integrity (ZO-1, Occludin, Claudin-1, and MUC2). Consequently, these mechanisms reduce diarrhea incidence and enhance growth performance in weaned piglets.

## 2. Materials and Methods

Main Instruments and Equipment:

Agilent 1260 Infinity High-Performance Liquid Chromatograph (HPLC) (Agilent Technologies, USA); Diode Array Detector (DAD) (Agilent Technologies, USA); Rotary Evaporator (Shanghai Yarong Biochemical Instrument Factory, China); KDM Thermostatic Heating Mantle (Yongxing Instrument Factory, Hancheng, Shandong Province, China); 0.22 μm Organic Phase Syringe Filters (Biosharp Life Sciences, China); TG16 Tabletop High-Speed Centrifuge (Shanghai Luxiangyi Centrifuge Instrument Co., Ltd., China); Electric Thermostatic Incubator (Shanghai Yuejin Medical Instrument Co., Ltd., China); Vacuum Freeze Dryer (Shanghai Yuming Instrument Co., Ltd., China).

### 2.1. Preparation of CAG

Following the standard preparation method for traditional Chinese medicine, begin by taking *Codonopsis pilosula* (Dangshen) (20 g), *Astragalus membranaceus* (Huangqi) (20 g), and *Glycyrrhiza uralensis* (Gancao) (10 g) and crushing them into a fine powder. Add ten times the volume of distilled water to the mixture and allow it to soak for 30 min. Subsequently, decoct the mixture for one hour, repeating this decoction process twice. After filtration, add ten times the volume of ethanol to the residue and decoct it for an additional hour. Combine the filtrates from the three extractions. Concentrate the combined filtrate using a rotary evaporator under reduced pressure until it adheres to the container walls. Finally, lyophilize the concentrated extract using a vacuum freeze dryer to obtain the CAG, which should be stored at 4 °C [27].

### 2.2. Determination of CAG Components

The determination of polysaccharide and flavonoid contents in the CAG was conducted using the phenol-concentrated sulfuric acid method and the sodium nitrite–aluminum nitrate method, respectively. Concurrently, the contents of seven key index components, namely Calycosin-7-glucoside, lobetyolin, liquiritin, ononin, calycosin, liquiritigenin, and formononetin, were analyzed using high-performance liquid chromatography (HPLC). Chromatographic separation was achieved using an Agilent ZORBAX SB-C18 analytical column (250 mm × 4.6 mm, 5 μm particle size) with binary gradient elution (Agilent Technologies, USA). The mobile phase comprised (A) aqueous 0.1% (*v*/*v*) H_3_PO_4_ and (B) chromatographic-grade acetonitrile. Chromatographic separation was conducted under optimized parameters using a triphasic elution protocol. The mobile phase composition progressed through three distinct stages: an initial 15 min phase with solvent B increasing from 10% to 35%, followed by a 10 min linear gradient elevating B concentration from 35% to 55%, culminating in a final 10 min isocratic phase at 67% B. Operational conditions included a constant 0.7 mL/min mobile phase flow through the thermostated column maintained at 25 °C. Analytical detection was performed at 254 nm wavelength with standardized 20 μL sample loading per injection.

### 2.3. Animals, Experimental Design, and Management

Eighty 28-day-old weaned piglets (Large White × Long White × Duroc), with an average body weight of 7.78 ± 0.074 kg, were randomly assigned to four groups: Control, LCAG (0.1% CAG), MCAG (0.5% CAG), and HCAG (1.0% CAG). The trial lasted for 28 days. Each group had an equal number of male and female piglets. The trial lasted 28 days, during which time all piglets were fed a basal diet based (Table 1). The chemical composition of the basal diet was determined using FOSS NIRS 2500 (FOSS Analytical, Denmark) with a scanning range of 400–2498 nm at 0.5 nm resolution. Spectra were interpreted via Dairy One Forage Lab Database (Ithaca, NY, USA; version 2023.1)

All piglets were housed in environmentally controlled facilities with ad libitum access to feed and water throughout the 28-day trial. Body weight was measured for each piglet after an overnight fast on the morning of day 1 (trial initiation) and day 29 (trial termination). Daily feed intake per group was recorded throughout the experimental period. Average daily gain (ADG), average daily feed intake (ADFI), and feed-to-gain ratio (F/G) were calculated based on data collected during the trial. Additionally, instances of diarrhea were systematically documented and assigned scores based on severity: 0 indicated normal stools, 1 denoted loose stools, 2 represented partial diarrhea, 3 corresponded to diarrhea, and 4 signified severe watery diarrhea.

Average Daily Feed Intake (ADFI):

1. At 07:30 daily, residual feed was collected, oven-dried, and weighed (±0.1 g).
2. Daily feed provision = Previous residual + 1.2 × estimated daily consumption.
3. Feed offered three times daily (08:00, 13:00, 18:00)
4. Daily intake = Total offered − Next day’s dried residual.
5. ADFI (g/pig/day) = Σ(29-day intake)/(20 pigs × 29 days).

On day 29 of the trial, venous blood was collected from the anterior vena cava of piglets in each experimental group. Serum was isolated by centrifugation (3000× *g*, 15 min) and subjected to comprehensive analysis of biochemical profiles, antioxidant status, and immune biomarkers. Based on the evaluation of growth performance, diarrhea incidence, and serum indices, piglets from the 1.0% CAG (CAG) were selected for autopsy to further investigate intestinal health. In both the Control and CAG groups, 8 pigs were randomly chosen and euthanized via intramuscular injection of sodium pentobarbital (40 mg/kg BW). The small intestine was flushed with saline, and sections from the middle of the jejunum and ileum were excised. Portions of these sections were fixed in 10% neutral formaldehyde for morphological analysis, while others were frozen for subsequent studies. Additionally, cecal contents were collected and stored at −80 °C.

### 2.4. Serum Immunoglobulin and Inflammatory Factor Levels

Serum immunoglobulin (Ig) and serum inflammatory factor levels were assessed using ELISA kits, specifically measuring IgA (Beijing Solarbio, Cat#SEKP-0013), IgM (Beijing Solarbio, Cat#SEKP-0014), IL-1β (Beijing Solarbio, Cat#SEKP-0001), TNF-α (Beijing Solarbio, Cat#SEKP-0009), and IL-10 (Beijing Solarbio, Cat#SEKP-0007). The procedures were conducted in accordance with the instructions provided by the kit manufacturers.

### 2.5. Scanning Electron Microscopy

Ileal tissue observed by scanning electron microscope (SEM) was fixed with 1% osmium acid for 1–2 h, dehydrated by alcohol gradient, then tissue conductivity was processed, and the samples were subjected to image acquisition by scanning electron microscope JSM-IT700HR produced by Japan Electronics Corporation (JEOL).

### 2.6. Hematoxylin and Eosin (HE) Staining

Then, 10% neutral formaldehyde-fixed jejunum and ileum tissues, gradient alcohol dehydration, paraffin embedding, tissue wax blocks were cut into 5 μm thick sections, hematoxylin and eosin stained, and neutral tree resin sealed the sections. Image acquisition of the sections was performed using a slide scanning imaging system (Shenzhen Shengqiang Technology Co., Ltd., SQS-600P). Morphometric analysis of intestinal segments included quantification of villus height (VH) and crypt depth (CD) using ImageJ (v1.53, NIH). The villus height-to-crypt depth ratio (VCR) was subsequently derived from these measurements.

### 2.7. Tissue PAS Staining

Sections of 5 µm of jejunum and ileum tissues were placed on slides, oxidized by adding PAS oxidant for 5 min, stained with Schiff reagent for 10 min, stained with hematoxylin staining solution for nuclei for 3 min, and the sections were sealed with neutral dendrimer.

### 2.8. RT-qPCR

Jejunal tissue samples underwent RNA isolation employing a specialized RNA extraction system (TransGen Biotech, ET101-01-V2). Spectrophotometric quantification at 260/280 nm absorbance ratios verified nucleic acid purity and concentration profiles. Reverse transcription was performed using a commercial reverse transcription master mix (Accurate Biotechnology AG11728) following standardized protocols. The synthesized cDNA products were cryopreserved at −80 °C prior to downstream RT-qPCR analyses. The primers for RT-qPCR, as detailed in Table 2, were designed using the NCBI website.

### 2.9. Western Blotting

Proteins from jejunal tissue were extracted, and their concentrations were determined using a BCA kit (Solarbio, Cat#PC0020). The extracted proteins were then separated by SDS-PAGE electrophoresis. For protein blot analysis, primary antibodies against ZO-1 (Bioss, bs-1329R, 1:1000), Occludin (Proteintech, 13409-1-AP, 1:4000), Claudin1 (Proteintech, 13050-1-AP, 1:1000), MUC2 (Abcam, ab-272692, 1:700), and GAPDH (Proteintech, 10494-1-AP, 1:5000) were employed. Secondary HRP-coupled anti-rabbit antibodies (Proteintech, SA00001-2) were used at a dilution of 1:10,000. Visualization was conducted using an Amersham ImageQuant 800 chemiluminescence instrument (Cytiva, USA), and protein quantification was performed with Image J software.

### 2.10. Levels of SCFAs in Cecum Contents

To prepare the internal standard dilution, 20 μL of n-butanol was combined with 980 μL of water and thoroughly mixed. Subsequently, 100 μg of cecum contents were precisely weighed and mixed with 500 μL of water. Two steel beads were added, and the mixture was homogenized at 50 Hz for 900 s. Following homogenization, the mixture was centrifuged at 41,200× *g* for 10 min at 4 °C. The resulting supernatant was filtered through a 0.22 μm filter. From this filtered solution, 198 μL was extracted, to which 2 μL of the n-butanol internal standard diluent was added, and the mixture was thoroughly mixed. For the on-line testing, 1 μL of this prepared sample was used.

#### Chromatographic Conditions

Chromatographic separation was conducted using an Agilent J&W GC column (DB-FFAP, 30 m × 0.25 mm, 0.25 μm film thickness) with a programmed thermal protocol. The injection port temperature was stabilized at 220 °C throughout the analysis. A multi-stage temperature gradient was implemented: initial 2 min equilibration at 60 °C occured, followed by a 10 °C/min ramp to 120 °C with 3 min isothermal hold, then a final heating phase reaching 180 °C for 5 min stabilization. Sample introduction employed split-mode injection (30:1 split ratio) with manual syringe delivery of 1 μL aliquots. Ultrapure nitrogen (>99% purity) served as carrier gas under a constant 1 mL/min flow regime. Compound detection was achieved using an FID detection system maintained at 280 °C.

### 2.11. Data Analysis

Statistical analyses were performed using IBM SPSS Statistics 26 (IBM Corp., Armonk, NY, USA). After confirming normality (Shapiro–Wilk test) and homogeneity of variance (Levene’s test). Univariate ANOVA with Tukey’s post hoc test was performed for multi-group comparisons (e.g., dose–response groups). Independent *t*-tests were used for two-group comparisons (e.g., Control vs. CAG). Significance was defined at *p* < 0.05 (*) and *p* < 0.01 (**). Results are presented as mean ± SEM. Graphical outputs were generated with GraphPad Prism 8 and refined in Adobe Photoshop 2023 using non-destructive editing. Protein band quantification used ImageJ v1.53 (NIH, MD, USA) with background subtraction.

## 3. Results

### 3.1. Component Analysis of CAG

**Figure 1:**
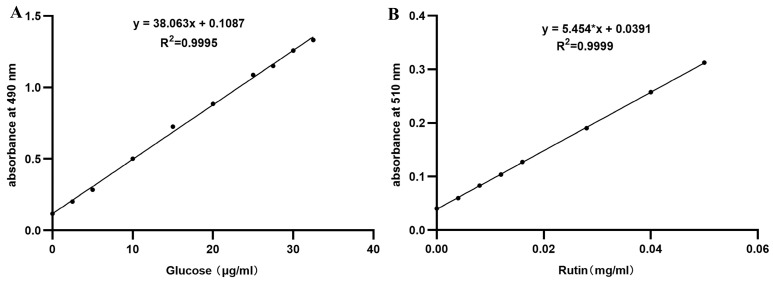
Schematic diagram of the standard curve for glucose and rutin. (**A**) Standard curve for glucose. (**B**) Standard curve for rutin.

**Figure 2:**
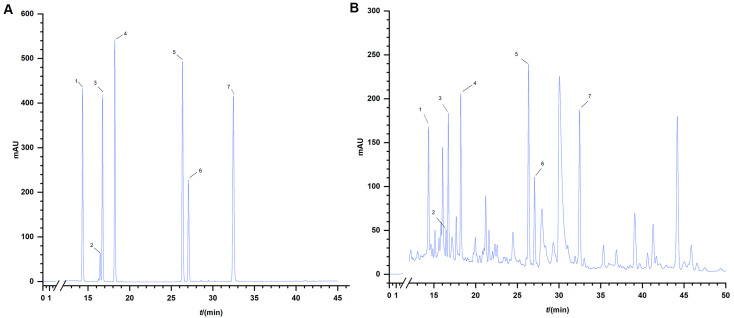
HPLC chromatogram of 6 components (custom-rendered). (**A**) HPLC chromatogram of mixed standards. (**B**) HPLC chromatogram of six components in CAG. (1) Calycosin-7-glucoside. (2) Lobetyolin. (3) Liquiritin. (4) Ononin. (5) Calycosin. (6) Liquiritigenin. (7) Formononetin.

The standard curves for anhydrous glucose and rutin, illustrated in Figure 1, respectively, were employed to calculate and determine the contents of polysaccharides and flavonoids in CAG using their corresponding linear equations. The polysaccharide and flavonoid contents in CAG were found to be 694.1 mg/g and 11.689 mg/g, respectively. Figure 2 displays the chromatograms of the mixed standards and CAG, indicating a satisfactory separation of the index components, with a smooth baseline and well-corresponding retention times between the Control and the samples. The concentrations of various compounds in CAG, including Calycosin-7-glucoside, lobetyolin, liquiritin, ononin, calycosin, liquiritigenin, and formononetin, were determined to be 19.8 μg/mL, 23.1 μg/mL, 86.8 μg/mL, 14.2 μg/mL, 16.4 μg/mL, 35.7 μg/mL, and 17.2 μg/mL, respectively, as presented in Table 3.

### 3.2. CAG Improves Growth Performance of Weaned Piglets

Table 4 demonstrates that, despite no significant difference in IBW between groups (*p* > 0.05), the inclusion of CAG in the basal diet markedly enhanced the FBW (*p* < 0.05) and ADG (*p* < 0.05) of weaned piglets. Furthermore, the addition of CAG increased the ADFI, reduced the F/G, and lowered the incidence of diarrhea compared to the Control group, with the most pronounced effects observed in the HCAG group. These findings indicate that CAG effectively promotes the growth performance of piglets experiencing the stress of weaning. Although the ADFI did not exhibit a significant change, the economic efficiency can be improved by reducing the F/G.

### 3.3. CAG Regulates Serum Levels of Immunoglobulins and Inflammatory Factors

The study found that compared to the Control group, CAG supplementation at concentrations of 0.1% to 1.0% significantly increased the lgA levels (*p* < 0.05), while HCAG notably elevated the lgM levels (*p* < 0.01). These findings suggest that CAG enhances the immunity of weaned piglets and boosts their disease resistance capabilities. Furthermore, MCAG significantly decreased the IL-1β levels (*p* < 0.01), although CAG did not have significant effects on IL-10 and TNF-α levels. It is important to note that IL-1β and TNF-α are pro-inflammatory cytokines, whereas IL-10 functions as an anti-inflammatory cytokine. Thus, the levels of these inflammatory factors are indicative of the overall health status of the piglets. The results imply that CAG effectively mitigates intestinal inflammation induced by weaning stress in piglets, as illustrated in Table 5.

Evaluating various parameters such as growth performance, diarrhea rate, serum biochemical indices, antioxidant indices, and immune indices, the study identified the HCAG as the optimal dose. Consequently, piglets in the HCAG group were chosen for further investigation to assess the impact on the intestinal barrier.

### 3.4. CAG Regulation of SCFAs Levels in Weaned Piglets

**Figure 3:**
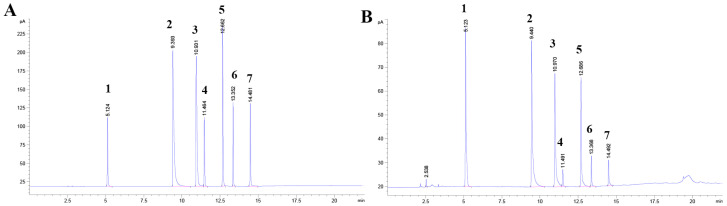
GC of SCFAs in cecum contents (instrument-software export). (**A**) GC of mixed standards. (**B**) GC of the contents of the cecum. (1) N-butanol (internal standard). (2) Acetic acids. (3) Propionic acids. (4) Butyric acids. (5) Isobutyric acid. (6) Valeric acids. (7) Isovaleric acids.

The gas chromatograms of both the mixed Control and the cecum contents displayed well-separated components with a smooth baseline, and the retention times of the Control and the samples corresponded accurately (Figure 3). The inclusion of CAG in the diet significantly elevated the concentrations of acetic, propionic, butyric, and isobutyric acids in the cecum contents of weaned piglets compared to the Control group. Notably, acetic, propionic, and butyric acids comprised 90% of the total acids. In contrast, there was no significant difference in the levels of valeric and isovaleric acids (Table 6).

### 3.5. CAG Improves Intestinal Barrier in Weaned PIglets

**Figure 4:**
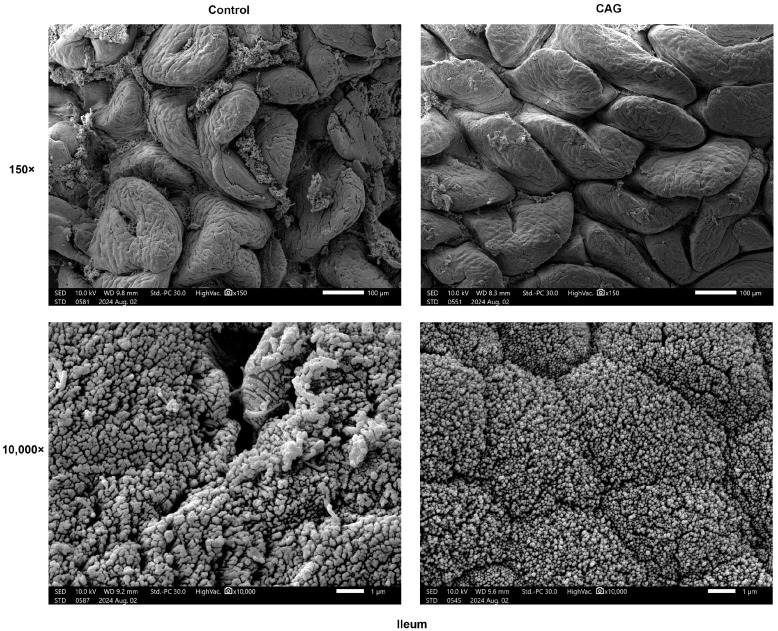
Morphology of the ileum by scanning electron microscopy. Control, the control group, where piglets were fed the basal diet; CAG, the CAG group, where piglets were fed the basal diet supplemented with 1.0% CAG. *n* = 3.

**Figure 5:**
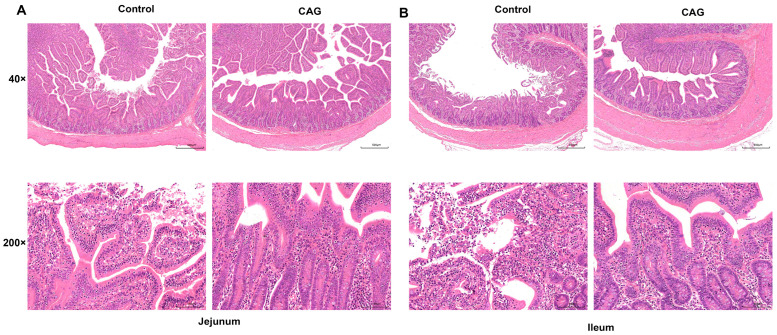
Morphology of jejunum and ileum observed by H&E staining. (**A**) H&E staining of the jejunum. (**B**) H&E staining of the ileum. Control, the control group, where piglets were fed the basal diet; CAG, the CAG group, where piglets were fed the basal diet supplemented with 1.0% CAG. *n* = 3.

To assess the impact of CAG treatment on epithelial morphology damage in the small intestine of weaning-stressed piglets, SEM and H&E staining were utilized. SEM images at 150× magnification demonstrated that CAG treatment reduced surface damage to the ileal villi. Furthermore, at a magnification of 10,000×, CAG was observed to increase the number of microvilli, resulting in a neater and denser microvilli morphology (Figure 4). H&E staining revealed that, in the Control group, the jejunum and ileum tissues exhibited necrosis and shedding of intestinal villi, with villi breaking off and detaching, leading to a loss of epithelial structure in the mucosa. Additionally, there was a small amount of inflammatory cell infiltration, predominantly lymphocytes, which was significantly ameliorated in the CAG group (Figure 5). As presented in Table 7, CAG treatment led to a significant increase in the VH of the ileum compared to the Control group (*p* < 0.01), and there was a significant difference in the CD of both the jejunum and ileum between the Control and CAG groups *(p* < 0.05). The VCR in the jejunum and ileum of the weaned piglets was significantly elevated following CAG treatment (*p* < 0.01). These findings indicate that CAG effectively mitigates surface damage to small intestinal villi and repairs intestinal damage induced by weaning stress in piglets.

**Figure 6:**
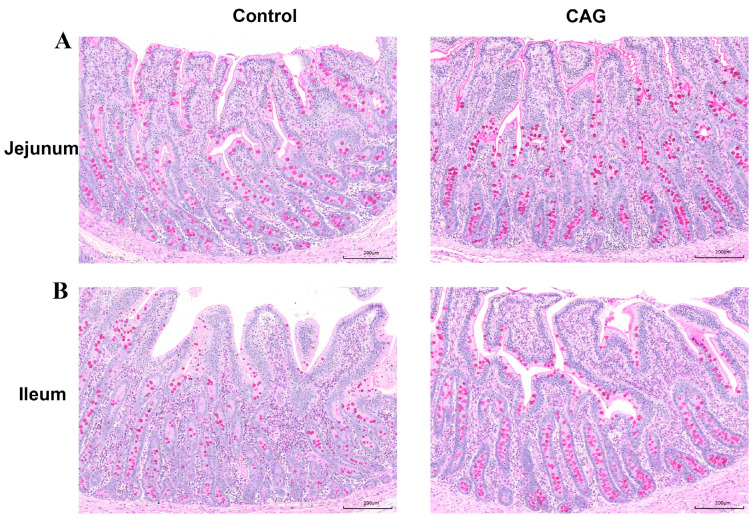
PAS staining of the jejunum and ileum. (**A**) PAS staining of the jejunum. (**B**) PAS staining of the ileum. Control, the control group, where piglets were fed the basal diet; CAG, the CAG group, where piglets were fed the basal diet supplemented with 1.0% CAG. Periodic Acid-Schiff stain (PAS). *n* = 3.

**Figure 7:**
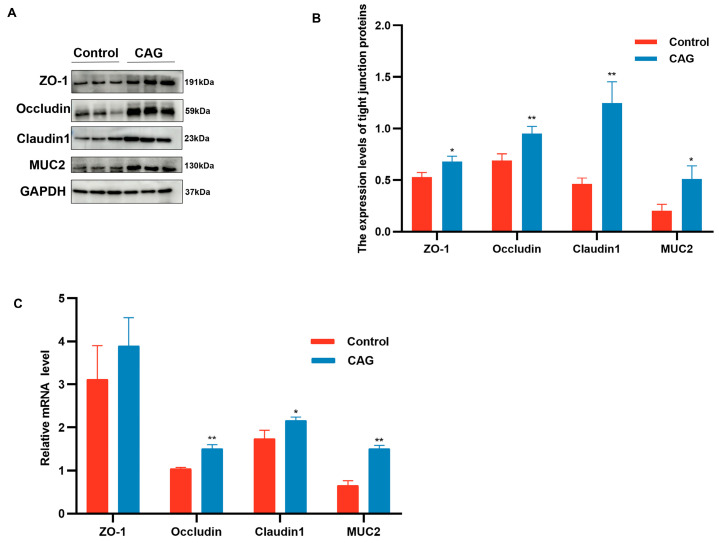
Expression of jejunal tight junction-associated proteins and genes analyzed by Western blot and RT-qPCR. (**A**) Representative bands for Western blot. (**B**) Western blot of tight junction proteins and MUC2 in jejunum tissue. (**C**) mRNA expression of ZO-1, Occudin, Claudin1, MUC2 in jejunal tissue. Control, the control group, where piglets were fed the basal diet; CAG, the CAG group, where piglets were fed the basal diet supplemented with 1.0% CAG. Glyceraldehyde-3-Phosphate Dehydrogenase (GAPDH); Zonula Occludens-1 (ZO-1); Mucin 2 (MUC2). Data were shown as means ± SEM (*n* = 6), *, *p* < 0.05; **, *p* < 0.01.

The impact of CAG on intestinal goblet cells in weaned piglets was assessed through PAS staining of the jejunum and ileum. As illustrated in Figure 6 and Table 8, CAG treatment significantly increased the number of goblet cells in both the jejunum and ileum compared to the Control group (*p* < 0.01). Goblet cells are responsible for mucus secretion, which serves to lubricate and protect the intestinal mucosa. Consequently, the increase in goblet cell numbers due to CAG treatment likely promoted mucus secretion, thereby enhancing the intestinal mucosal barrier and preventing pathogen invasion. Furthermore, compared to the Control group, CAG significantly upregulated the protein expression of jejunal tight junction proteins ZO-1, Occludin, Claudin1, and mucin MUC2 (Figure 7A,B). It also significantly increased the mRNA expression of Occludin, Claudin1, and MUC2, although it did not significantly affect the mRNA expression of MUC2 (Figure 7C). These findings confirm that CAG improves barrier function, mitigates weaning stress-induced reductions in tight junctions, and protects the piglet intestine from stress-induced epithelial damage.

## 4. Discussion

In this study, CAG demonstrated its anti-inflammatory, antioxidant, and immune-enhancing functions through various components, including polysaccharides, flavonoids, Calycosin-7-glucoside, lobetyolin, liquiritin, ononin, calycosin, liquiritigenin, and formononetin. These constituents collectively contributed to alleviating stress, reducing diarrhea incidence, and improving growth performance in weaned piglets. Specifically, *Codonopsis pilosula* polysaccharides and *Astragalus membranaceus* polysaccharides have been shown to possess immunomodulatory, anti-inflammatory, antioxidant, and antiviral properties [28,29]. Flavonoids, on the other hand, exhibit multiple pharmacological effects, such as bacteriostatic, anticancer, antioxidant, anti-inflammatory, and antiviral activities [30]. The previous literature has reported that herbal medicines and their combinations containing these components enhance intestinal antioxidant capacity and modulate colonic microbiota in weaned pigs [31], findings that are consistent with the results of the present study.

The integrity of intestinal structure is crucial for nutrient absorption in piglets [32]. Intestinal morphology, which encompasses VH, CD, and the VCR, serves as an important indicator of intestinal health and absorption efficiency. Optimal intestinal function and improved digestive and absorptive capabilities are characterized by increased VH and VCR, as well as reduced CD [33]. Numerous studies have demonstrated that weaned animals frequently experience intestinal villi shedding or damage, crypt layer hypertrophy, and mucosal thinning, which collectively compromise barrier integrity and nutrient assimilation capacity [34,35]. For instance, Bomba et al. observed that VH and VCR were significantly lower on day 5 after weaning compared to pre-weaning levels [36]. Hu et al. found that weaning causes lasting structural changes in piglets’ small intestines, with the jejunum’s VH significantly lower on day 15 post-weaning compared to pre-weaning [37]. Consistent with these findings, the present study demonstrated that early weaning causes intestinal morphological damage in piglets, including increased CD, decreased VH, and reduced VCR. However, treatment with CAG significantly ameliorated these adverse effects by increasing jejunal and ileal VH and VCR, reducing CD, decreasing inflammatory cells, and improving small intestinal villous surface damage, thereby maintaining the integrity of the intestinal barrier.

The gastrointestinal system serves as the primary interface for nutrient assimilation and xenobiotic surveillance, fulfilling dual critical functions in substrate metabolism and defense against pathogenic invasion. This homeostatic equilibrium fundamentally relies on the structural and functional integrity of the gastrointestinal defense system [38]. The gut’s protective architecture comprises four interactive defense strata: epithelial tight junction complexes, antimicrobial biochemical secretions, gut-associated lymphoid tissue, and commensal microbiota networks, which synergistically maintain intestinal microenvironmental stability through multi-layered crosstalk [39,40]. When piglets are separated from their mothers, the intestinal defense barriers suffer irreversible damage, leading to increased intestinal permeability and infiltration of inflammatory factors. Research indicates that the earlier a piglet is weaned, the more persistent the intestinal damage [41]. Intestinal barrier integrity is primarily governed by tight junctions (TJs)—specialized multiprotein assemblies localized at the apicolateral membrane domains of enterocytes. These complexes incorporate transmembrane components (e.g., Claudin, Occludin) and cytoplasmic scaffolding proteins such as zonula occludens-1 (ZO-1). TJs critically regulate paracellular permeability and preserve the structural continuity of the intestinal barrier Weaning stress has been shown to disrupt various tight junction proteins, including Claudin1, Occludin, ZO-1, ZO-2, and ZO-3, potentially through the MAPK and TGF-b1 signaling pathways [42,43,44]. The study found that curcumin increased the protein expression of ZO-1 and Claudin-1 in the jejunum of piglets under oxidative stress [45]. Aligned with existing evidence, our data establish that CAG administration substantially elevates jejunal protein abundance (ZO-1, Occludin, and Claudin-1) and potently induces transcriptional upregulation of Occludin and Claudin-1 genes. Collectively, these coordinated responses fortify intestinal barrier integrity at both translational and transcriptional levels.

The chemical defense system of the gut is fundamentally constituted by a mucus-based matrix, primarily containing goblet cell-derived mucins (MUC family) and epithelium-secreted antimicrobial factors [46]. Among the mucins, MUC2 is the predominant component of intestinal mucus, playing a crucial role in intestinal lubrication, resistance to pathogenic bacteria, and intercellular signaling [47]. Weaning stress has been reported to impair the differentiation of mucus-producing goblet cells, resulting in decreased mucin secretion [48]. Early weaning reduces intestinal mucin secretion and alters mucin glycosylation, weakening the intestinal chemical barrier and increasing the risk of infections [49]. Congruent with prior evidence, weaning stress induces significant downregulation of MUC2 transcription in piglets. This gene suppression directly compromises intestinal chemical barrier function through impaired mucin biosynthesis and aberrant secretory dynamics [50]. In the present study, CAG treatment significantly enhanced the goblet cell population in the jejunum and ileum and led to a marked increase in both jejunal MUC2 protein levels and MUC2 gene expression. A decrease in intestinal mucin secretion thins the mucus layer of the intestinal mucosa, allowing pathogenic microorganisms to penetrate more easily and disrupt the intestinal chemical barrier. Therefore, maintaining normal mucin secretion and expression is essential for preserving intestinal barrier function.

The intestinal immune barrier, a sophisticated and intricate localized immune system, comprises primarily immune organs, immune cells, and immune molecules [51]. It plays a crucial role in recognizing exogenous antigenic stimuli while preventing the animal body from becoming hypersensitive to harmless antigens [52]. Porcine intestinal immunity achieves functional maturation around postnatal week 7. Current intensive farming practices, however, implement weaning at 3–4 weeks—a developmental stage characterized by immature gut-associated lymphoid tissue (GALT) and compromised immunobarrier competence. This premature weaning induces stress, disrupting the immune barrier function and increasing susceptibility to diseases [53]. Weaning-induced stress triggers hyperactivation of the gut immune network, provoking dysregulated overexpression of pivotal pro-inflammatory mediators (TNF-α, IFN-γ, IL-1β, IL-6, IL-8). This cytokine storm ultimately drives structural and functional compromise of the intestinal milieu [54,55,56,57]. Pro-inflammatory cytokines including TNF-α, IL-1β, and IL-10 play pivotal roles in inflammatory cascades by promoting cellular damage and perpetuating inflammatory responses, thereby exacerbating oxidative stress. Consequently, monitoring a compound’s modulatory effects on inflammatory cytokine expression serves as a critical indicator for evaluating its efficacy in mitigating oxidative stress. Diquat-induced oxidative stress significantly elevates IL-1β levels in piglets compared to controls, while hydroxytyrosol treatment effectively suppresses IL-1β expression [58]. Stress conditions deplete serum IgM levels in piglets, whereas dietary supplementation with 1–5% Mulberry leaf restores IgM concentrations [4]. Limosilactobacillus reuteri SLZX19–12 substantially reduces ileal IL-1β gene expression in weaning-stressed piglets, indicating potent anti-inflammatory activity [59]. This study’s findings align with previous research, demonstrating that CAG enhances the secretion of IgA, IgM, and the anti-inflammatory factor IL-10, while inhibiting the secretion of pro-inflammatory factors TNF-α and IL-1β. In this study, IL-1β levels were significantly reduced in piglets receiving MCAG treatment, while remaining stable in the HCAG group. Notably, supplementation with 0.2–0.8% fermented Astragalus consistently enhanced serum immune parameters and growth performance in weaned piglets, with the 0.6% group demonstrating optimal efficacy—a finding consistent with our IL-1β data. This dose–response pattern may stem from the hormetic properties of bioactive compounds (e.g., Astragalus polysaccharides, glycyrrhizin from Glycyrrhiza uralensis), where high-dose supplementation potentially exceeds intestinal absorption capacity in piglets, thereby reducing systemic bioavailability. Concurrently, HCAG may have activated counter-regulatory pathways (e.g., through elevated IL-10 or TGF-β), which modulated IL-1β suppression. Thus, higher dosages do not invariably confer superior therapeutic outcomes. These observations suggest that CAG may effectively mitigate weaning stress-induced intestinal inflammation and damage to the intestinal immune barrier in piglets.

SCFAs are produced through the fermentation of indigestible carbohydrates, such as dietary fiber, by intestinal probiotics like Lactobacillus and Bifidobacterium [60]. SCFAs play a crucial role in providing oxidative energy, regulating blood glucose levels, and maintaining water–electrolyte balance through hybridization with monosaccharide molecules. Current evidence indicates butyric acids serves as the primary energy substrate for colonocytes, providing 70–80% of their metabolic requirements through β-oxidation [61]. Propionic acids are primarily metabolized in the liver [62], while acetic acids enter systemic circulation for peripheral tissue utilization [63]. Therefore, butyric acid is more critical for the energy supply of epithelial cells. Additionally, they possess antimicrobial and anti-inflammatory properties, contribute to the regulation of intestinal flora balance, enhance intestinal function, modulate immune responses, exhibit anti-tumor effects, and influence gene expression [64,65]. Research has demonstrated that incorporating Mulberry leaf powder into feed at concentrations ranging from 1% to 5% significantly increases SCFAs levels in the cecum, particularly butyric acid, which aligns with the current study’s findings [4]. Butyric acid specifically promotes epithelial cell proliferation and tight junction synthesis by activating the Akt/mTOR signaling pathway [66,67]. Critically, SCFAs fulfill approximately 80% of the bioenergetic demand in colonic enterocytes. Concurrently, they reinforce the gut barrier through dual mechanisms: sustaining immunomodulatory activity of mucosal leukocytes and preserving the structural continuity of the epithelial lining [68]. Therefore, CAG enhances intestinal barrier function by increasing the concentrations of acetate, propionate, butyrate, and isobutyrate, which provide energy to the intestinal epithelium, inhibit the release of inflammatory factors, upregulate the expression of tight junction proteins (such as ZO-1, Occludin, and Claudin1), and thereby restore and strengthen the intestinal barrier.

In livestock research, growth performance (ADG, F/G, diarrhea rate) is the primary clinical endpoint. The 1.0% CAG group showed superior efficacy in all three key metrics, offering direct economic value. While 0.5% CAG optimized IgA and IL-1β, the 1.0% dose achieved significantly better IgM (a critical mucosal immunoglobulin) and growth performance without adverse effects (e.g., no increased diarrhea). The absence of linearity in TNF-α may relate to threshold effects (e.g., receptor saturation) or feedback regulation (e.g., high-dose anti-inflammatory activation). In summary, the 1.0% CAG dose was selected based on comprehensive production-oriented evaluation.

1.0% CAG increased colonic butyrate levels by 59.7%, IgA increased by 25.45%, IgM increased by 20.28%, and a positive correlation with the VH/CD ratio was shown. Analysis via HE staining, PAS staining, and SEM revealed preserved intestinal barrier architecture in the 1.0% CAG group. Concurrent upregulation of tight junction proteins (ZO-1, Occludin, Claudin1), MUC2 protein, and associated gene expression provides the mechanistic basis for the observed reduction in diarrhea rate. Barrier repair enhanced nutrient absorption, directly improving ADG and F/G.

It is recognized that this study also lacked long-term assessments. Although the present work establishes CAG’s effectiveness in enhancing intestinal health and growth metrics, subsequent investigations should prioritize combinatorial therapies integrating CAG with probiotics, and in vitro elucidation of the temporal mechanisms governing gut microenvironment modulation. CAG, at doses of 0.1–1.0%, was shown to enhance barrier function under these experimental conditions; however, its translational potential requires validation in commercial production settings.

## 5. Conclusions

In conclusion, CAG prevented diarrhea caused by weaning stress and improved the intestinal barrier function. CAG can reduce the diarrhea rate and improve the growth performance of piglets by lowering the level of inflammatory factors, improving immunity, promoting the production of SCFAs, repairing intestinal damage, and improving the intestinal barrier function.

Perspective: Although this study confirms the efficacy of CAG in mitigating weaning stress, future research should explore synergistic effects with probiotics to alleviate stress throughout the entire swine production cycle. Such investigations would enhance growth performance, enable longitudinal health monitoring, improve meat quality attributes of market-ready finisher pigs, and accelerate the agricultural application of CAG as a sustainable antibiotic alternative.

## Figures and Tables

**Table 1:** Composition of basal diets and nutrient levels (Dry Matter, %).

Item	Content
Ingredients	
Corn	55.0
Fishmeal	1.0
Expanded soybean	4.9
Soybean meal (46%)	15.2
Fermented soybean meal (50%)	8.1
Beer yeast powder	1.0
Wheat	4.0
Wheat bran	3.0
Soybean oil	0.5
Calcium hydrogen phosphate	1.2
Talcum powder	1.1
Sodium chloride	0.5
Lysine	2.0
Methionine	1.0
Threonine	1.5
Total	100
Energy and nutrient levels	
Crude protein	21.3
Crude fiber	5.0
Calcium	1.0
Total phosphorus	0.50
Neutral detergent fiber (MDF)	14.9
Acid detergent fiber (ADF)	7.50
OM	93.62
NE (Kcal/Kg)	2886
Fat	5.52

**Table 2:** Primer sequences of target genes selected for analysis by qRT-PCR.

Gene	Primer Sequences (5′–3′)
GAPDH	F: GTCGGAGTGAACGGATTTGGC R: GGAGGTCAATGAAGGGGTCA
ZO-1	F:ACCAGAAATACCTGACGGTGC R: GATGGCGTTACCCACAGCTT
Occludin	F: CAGGTGCACCCTCCAGATTG R: TGGACTTTCAAGAGGCCTGG
Occludin1	F: TCTTTCTTATTTCAGGTCTGGCT R: ACTGGGGTCATGGGGTCATA
MUC2	F: CCAGGTCGAGTACATCCTGC R: GTGCTGACCATGGCCCC

**Table 3:** Linear equations, linear range, and contents of the 7 components of CAG.

Components	Peak Time	Linear Regression Equation	Correlation Coefficient	Linear Range, μg/mL	Content, μg/mL
Calycosin-7-glucoside	14.346	Y = 65,174.87X + 19.85	0.999	6.0–48.0	19.8
Lobetyolin	16.463	Y = 8250.64X + 2.13	0.999	6.5–52.0	23.1
Liquiritin	16.741	Y = 13,007.68X + 30.51	0.999	29.0–232.0	86.8
Ononin	18.221	Y = 100,492.81X + 24.45	0.999	4.75–38.0	14.2
Calycosin	26.358	Y = 127,852.57X + 25.52	0.999	4.25–34.0	16.4
Liquiritigenin	27.076	Y = 25,221.09X + 13.98	0.999	9.87–79.0	35.7
Formononetin	32.469	Y = 106,533.80X + 84.61	0.999	5.1–41.0	17.2

**Table 4:** Effect of CAG on growth performance and diarrhea rate of weaned piglets.

Item	Control	LCAG	MCAG	HCAG	SEM	*p*-Value
IBW, kg	7.76	7.87	7.69	7.81	0.216	0.251
FBW, kg	18.66	19.26	19.40	20.54 **	0.661	0.047
ADG, g/d	375.86	392.78	403.98	438.72 **	19.319	0.015
ADFI, g/d	522.31	542.14	542.75	557.58	-	-
F/G	1.39	1.38	1.34	1.27	-	-
diarrhea rate, %	20.09	10.90	12.60	10.95	-	-

Control, the control group, where piglets were fed the basal diet; LCAG, the LCAG group, where piglets were fed the basal diet supplemented with 0.1% CAG; MCAG, the MCAG group, where piglets were fed the basal diet supplemented with 0.5% CAG; HCAG, the HCAG group, where piglets were fed the basal diet supplemented with 1.0% CAG. Initial body weight (IBW); final body weight (FBW); average daily gain (ADG); average daily feed intake (ADFI); feed-to-weight ratio (F/G). Data were shown as means ± SEM, SEM = Standard error of the mean (intra-group) (*n* = 16), **, *p* < 0.01.

**Table 5:** Serum levels of immunoglobulins and inflammatory factors.

Item	Control	LCAG	MCAG	HCAG	SEM	*p*-Value
IgA, ng/mL	0.82	1.15 **	1.24 **	1.10 **	0.041	0.000
IgM, ng/mL	118.83	117.36	118.62	149.06 **	5.133	0.017
IL-1β, pg/mL	812.67	889.57 *	692.13 **	757.00	35.128	0.003
IL-10, pg/mL	104.84	112.31	108.28	108.26	4.042	0.843
TNF-α, pg/mL	10.31	11.14	9.76	9.85	0.535	0.106

Control, the control group, where piglets were fed the basal diet; LCAG, the LCAG group, where piglets were fed the basal diet supplemented with 0.1% CAG; MCAG, the MCAG group, where piglets were fed the basal diet supplemented with 0.5% CAG; HCAG, the HCAG group, where piglets were fed the basal diet supplemented with 1.0% CAG. Data were shown as means ± SEM, SEM = Standard error of the mean (intra-group) (*n* = 8), *, *p* < 0.05; **, *p* < 0.01.

**Table 6:** The levels of SCFAs in cecum contents.

Item	Control	CAG	SEM	*p*-Value
Acetic acids, μmol/g	2.41	4.26 **	0.162	0.000
Propionic acids, μmol/g	1.02	1.47 *	0.107	0.014
Butyric acids, μmol/g	0.31	0.77 *	0.125	0.021
Isobutyric acid, μmol/g	0.017	0.069 *	0.012	0.013
Valeric acids, μmol/g	0.06	0.08	0.013	0.212
Isovaleric acids, μmol/g	0.05	0.06	0.009	0.717

Control, the control group, where piglets were fed the basal diet; CAG, the CAG group, where piglets were fed the basal diet supplemented with 1.0% CAG. Gas chromatogram (GC); short-chain fatty acids (SCFAs). Data were shown as means ± SEM, SEM = Standard error of the mean (intra-group) (*n* = 6), *, *p* < 0.05; **, *p* < 0.01.

**Table 7:** Jejunal and ileal villus height (VH), crypt depth (CD), and VH:CD ratio in weaned piglets.

Item	Control	CAG	SEM	*p*-Value
Jejunum				
VH, μm	435.0	514.2	37.98	0.056
CD, μm	226.8	197.5 *	10.34	0.013
VH:CD	1.9	2.6 **	0.14	0.000
Ileum				
VH, μm	385.1	521.1 **	21.37	0.000
CD, μm	235.6	201.0 *	11.91	0.013
VH:CD	1.6	2.6 **	0.14	0.000

Control, the control group, where piglets were fed the basal diet; the CAG group, where piglets were fed the basal diet supplemented with 1.0% CAG. Villus height (VH); crypt depth (CD); VH:CD (VCR). Data were shown as means ± SEM, SEM = Standard error of the mean (intra-group) (*n* = 3), *, *p* < 0.05; **, *p* < 0.01.

**Table 8:** Statistics on the number of goblet cells in the jejunum and ileum.

Item	Control	CAG	SEM	*p*-Value
Jejunum	135.00	217.88 *	22.126	0.020
Ileum	142.44	253.55 *	24.363	0.010

Control, the control group, where piglets were fed the basal diet; CAG, the CAG group, where piglets were fed the basal diet supplemented with 1.0% CAG. Periodic Acid-Schiff stain (PAS). Data were shown as means ± SEM, SEM = Standard error of the mean (intra-group) (*n* =3), *, *p* < 0.05.

## Data Availability

The data presented in this study are available upon request from the corresponding author (Y.L.-H).

## Abbreviations

The following abbreviations are used in this manuscript:

ADFI	Average daily feed intake
ADG	Average daily gain
CAG	Extraction of ShenQiGan
CD	Crypt depth
F/G	Feed-to-weight ratio
FBW	Final body weight
IBW	Initial body weight
